# The Influence of IL-10 and TNFα on Chondrogenesis of Human Mesenchymal Stromal Cells in Three-Dimensional Cultures

**DOI:** 10.3390/ijms150915821

**Published:** 2014-09-09

**Authors:** Michal Jagielski, Johannes Wolf, Ulrike Marzahn, Anna Völker, Marion Lemke, Carola Meier, Wolfgang Ertel, Owen Godkin, Stephan Arens, Gundula Schulze-Tanzil

**Affiliations:** Department for Orthopedic, Trauma and Reconstructive Surgery, Charité-Universitätsmedizin Berlin, Campus Benjamin Franklin, 14195 Berlin, Garystrasse 5, Germany; E-Mails: michal.jagielski@charite.de (M.J.); tgjpw@web.de (J.W.); umarzahn@gmail.com (U.M.); anna.voelker@medizin.uni-leipzig.de (A.V.); marion.lemke@charite.de (M.L.); carola.meier@charite.de (C.M.); wolfgang.ertel@charite.de (W.E.); owen.godkin@gmail.com (O.G.); stephan.arens@charite.de (S.A.)

**Keywords:** bone-marrow MSC, three-dimensional culture, articular chondrocytes, chondrogenesis, IL-10, TNFα

## Abstract

Chondrogenic differentiated mesenchymal stromal cells (MSCs) are a promising cell source for articular cartilage repair. This study was undertaken to determine the effectiveness of two three-dimensional (3D) culture systems for chondrogenic MSC differentiation in comparison to primary chondrocytes and to assess the effect of Interleukin (IL)-10 and Tumor Necrosis Factor (TNF)α on chondrogenesis by MSCs in 3D high-density (H-D) culture. MSCs were isolated from femur spongiosa, characterized using a set of typical markers and introduced in scaffold-free H-D cultures or non-woven polyglycolic acid (PGA) scaffolds for chondrogenic differentiation. H-D cultures were stimulated with recombinant IL-10, TNFα, TNFα + IL-10 or remained untreated. Gene and protein expression of type II collagen, aggrecan, sox9 and TNFα were examined. MSCs expressed typical cell surface markers and revealed multipotency. Chondrogenic differentiated cells expressed cartilage-specific markers in both culture systems but to a lower extent when compared with articular chondrocytes. Chondrogenesis was more pronounced in PGA compared with H-D culture. IL-10 and/or TNFα did not impair the chondrogenic differentiation of MSCs. Moreover, in most of the investigated samples, despite not reaching significance level, IL-10 had a stimulatory effect on the type II collagen, aggrecan and TNFα expression when compared with the respective controls.

## 1. Introduction

Cartilage injury remains still an orthopedic challenge, since mature cartilage has only a limited capacity for intrinsic repair. A major restriction for cell-based strategies suitable for improving cartilage repair, such as matrix assisted chondrocyte transplantation (MACT), are limited in their accessibility of autologous cartilage for chondrocyte isolation and *in vitro* expansion as well as donor site morbidity. The clinical outcomes are still unsatisfying [1,2]. Mesenchymal stromal cells (MSCs) are an accessible cell source in the body capable for chondrogenic differentiation with low donor site morbidity and hence, could be a promising approach for articular cartilage repair. Osteochondral defects are usually covered by MSCs which emigrate from the bone marrow cavities into the defect and start chondrogenic differentiation [3].

However, effective, pure and permanent chondrogenic differentiation of MSCs still remains a challenge [4,5].

The role of particular cytokines in chondrogenic MSC differentiation is mostly unclear. Cartilage injury can lead to an inflammatory milieu and the development of osteoarthritis (OA) [6]. Pro-inflammatory cytokines such as Tumor Necrosis Factor (TNF)α play a crucial role in the pathogenesis of OA [7]. Whereas the chondrogenic differentiation of MSCs is inhibited by TNFα and Interleukin (IL)-1β involving the NF-κB pathway [8], TNFα is known to induce proliferation and migration of MSCs [9]. MSCs can easily be isolated, extensively and rapidly be expanded while maintaining their chondrogenic differentiation potential. Therefore, large cell numbers can be obtained for therapeutic use, whereby their immunosuppressive effects might be interesting in arthritis therapy [10].

MSCs exert their immunomodulatory effects by expressing IL-10 and furthermore enhancing its expression within local tissue [11,12,13]. IL-10 is a typical anti-inflammatory cytokine [14]. Its over-expression by MSC has been established as a tool to utilize their immunosuppressive potential, e.g., to suppress acute graft-*versus*-host disease [14,15]. Whether IL-10 is a valuable tool in OA therapy remains unclear.

It is crucial to develop an effective method of MSC cultivation that allows a pure differentiation towards the selected mesenchymal lineage. A challenging approach in chondrogenic differentiation of MSCs can be the use of 3D cultures. Most commonly used are the pellet cultures [16,17]. To optimize this process, some researchers developed different 3D cultures consisting of a cell aggregate on a porous membrane [4,18]. Another interesting approach to achieve chondrogenic differentiation is the application of MSCs on polyglycolic acid (PGA). PGA is a resorbable polymer that can already be found in clinical use [19,20]. PGA scaffolds may enhance the repair of cartilage defects and are able to induce chondrogenic differentiation of MSCs [21,22]. Several mediators such as Transforming Growth Factor (TGF)-β1 and -β3 are known to induce effective chondrogenic differentiation of MSCs [18,23,24,25].

The aim of the present study was to determine the effectiveness of a scaffold-free and a scaffold-associated 3D culture system for chondrogenic MSC differentiation in comparison to primary chondrocytes and to gain a first insight into the impact of IL-10 and TNFα on chondrogenic differentiation of MSCs.

## 2. Results

### 2.1. Results of Mesenchymal Stromal Cell (MSC) Characterization

**Figure 1:**
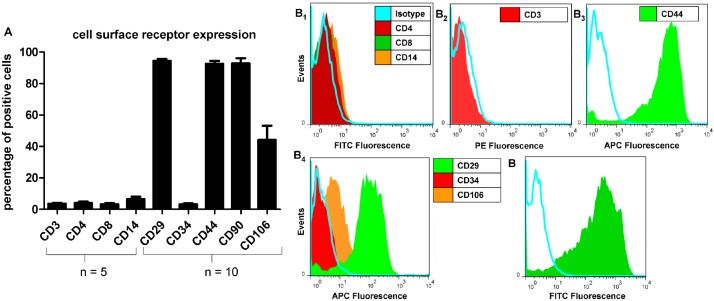
Characterization of mesenchymal stromal cells (MSCs) surface marker expression (passages 4–6) using flow cytometry. The isolated and adherent MSCs (cultured expanded for at least 4 passages) were tested for both, negative (CD3, CD4, CD8, CD14) (*n* = 5) and positive (CD29, CD44, CD90) markers (*n* = 10) using flow cytometry. The percentage of MSCs positive for the respective marker is shown (**A**); Less than a half of the MSCs were positive for the questionable surface marker CD106 (*n* = 10). The majority of the adherent MSCs was CD34 negative (*n* = 10) and negative for leukocyte surface proteins. The histograms of surface marker expression by MSCs of a representative donor are shown (**B_1_**–**B_5_**).

**Figure 2:**
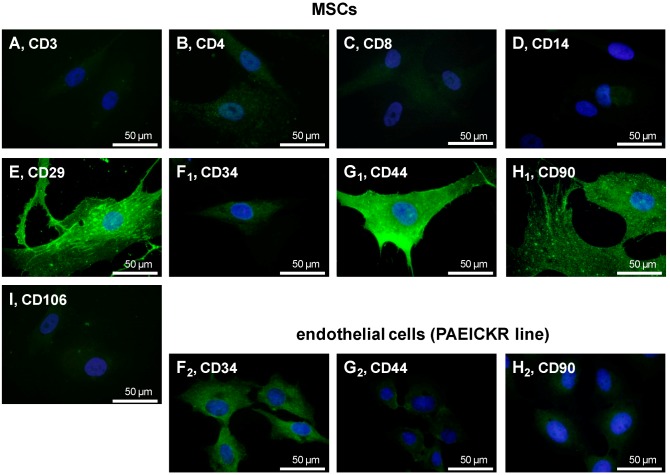
Characterization of MSCs surface marker expression (passages 4–6) using fluorescence microscopy. Surface markers such as leukocyte surface proteins CD3 (**A**); CD4 (**B**); CD8 (**C**); CD14 (**D**); cell adhesion protein CD29 (**E**); endothelial cell marker CD34 (**F_1_**,**F_2_**); hyaluronan receptor CD44 (**G_1_**,**G_2_**); mesenchymal cell marker CD90 (**H_1_**,**H_2_**); and CD106 (**I**) are depicted by immunofluorescence microscopy (green). Cells of the human endothelial cell line PAEICKR were immunolabelled for CD34 (**F_2_**), CD44 (**G_2_**) and CD90 (**H_2_**) as a control. Cell nuclei were counterstained using 4',6-diamidino-2-phenylindole (DAPI, blue). Scale bars = 50 µm.

More than 92% of isolated plastic adherent cells from all donors expressed CD29 (β1-integrin), CD44 (hyaluronan receptor) and CD90 (thymocyte differentiation antigen 1) and less than 8% expressed the leukocyte surface proteins CD3, CD4, CD8, CD14 and the hematopoetic cell marker CD34. The CD106 was expressed on 44.13% of all isolated cells (Figure 1). In agreement with the flow cytometrical results typical surface marker expression such as CD29, CD44 and CD90 could be depicted on the undifferentiated MSCs and localized using immunofluorescence labelling (Figure 2A–I). The CD34 expression on the MSCs was weak compared with the endothelial cell line (PAEICKR, positive control, Figure 2F_2_). The endothelial cell line expressed only very weakly CD44 and CD90 (Figure 2G_2_,H_2_).

### 2.2. Multipotency of MSCs

**Figure 3:**
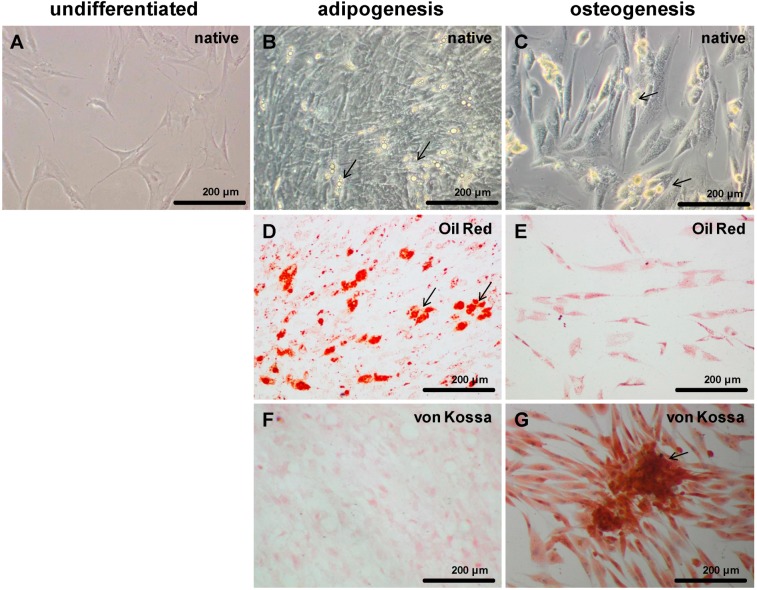
Adipogenic and osteogenic differentiated MSCs. Invert microscopical images of undifferentiated (**A**), adipogenic (**B**,**D**,**F**) and osteogenic (**C**,**E**,**G**) differentiated MSCs in monolayer culture. The cells were adipogenically and osteogenically differentiated for 21 days. Adipogenic differentiated cells revealed multiple fat vacuoles (**B**,**D**) which were red after oil red staining (**D**, arrows). Osteogenic differentiation of MSCs led to granulated elongated cells (**C**,**E**,**G**) which were von Kossa positive (**G**) and formed clusters of extracellular matrix deposits (**C**,**G**, arrows). Images of a representative experiment are shown. Scale bars = 200 µm.

Undifferentiated MSCs revealed a flattened and multipolar cell shape (Figure 3A). Chondrogenic differentiated cells formed 3D cell clusters when cultured in monolayer (not shown) and expressed typical cartilage markers such as type II collagen and sulfated glycosaminoglycans in H-D culture and PGA scaffold culture after 14 days. In contrast to undifferentiated MSCs (Figure 3A), adipogenically differentiated cells revealed multiple fat vacuoles after 21–28 days (Figure 3B–D) which were oil red positive (Figure 3D). Osteogenic differentiated cells became granulated and had a mostly bipolar shape (Figure 3C,E,G) compared to undifferentiated cells. Compared to adipogenic cells they were (Figure 3F) von Kossa positive (Figure 3G).

### 2.3. IL-10 and IL-10 Receptor-α Expression

**Figure 4:**
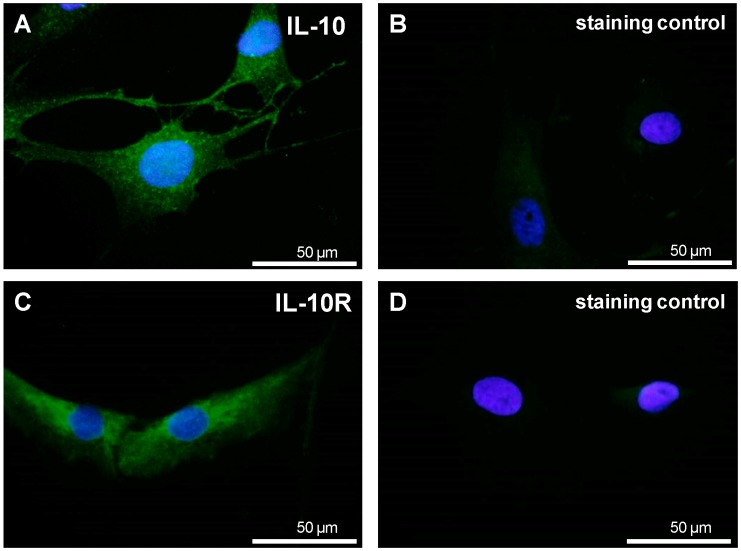
IL-10 and IL-10Rα expression in undifferentiated MSCs. Undifferentiated MSCs were cultured for at least 3 passages in monolayer culture and immunolabelled with IL-10 (**A**) or IL-10R (**C**) specific antibodies or respective isotype controls (**B**,**D**) and Alexa-488 coupled secondary antibodies (green). Cell nuclei were counterstained using DAPI (blue). A representative experiment of 4 independent tests with cells of different donors is shown. Scale bars = 50 µm.

Undifferentiated MSCs cultivated in monolayer culture for at least 3 passages revealed synthesis of IL-10. In addition IL-10 was detectable in the cytoplasm and in various cases in long cellular cytoplasmic extensions in cell-cell contact areas. The IL-10 receptor (IL-10R)α chain could also be detected using specific antibodies revealing a cytoplasmic distribution (Figure 4A–D). In contrast to IL-10Rβ chain which is also a component in other IL-10 family cytokine receptors the α chain is specific for IL-10 signaling.

**Figure 5:**
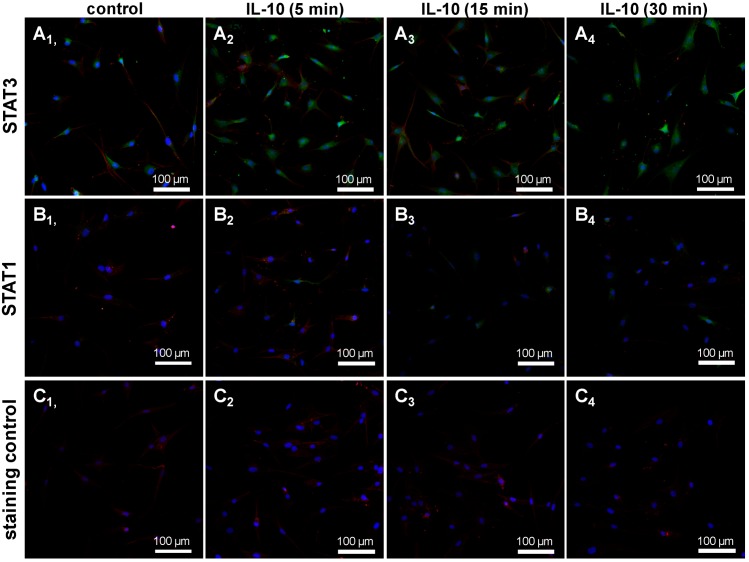
STAT3 and STAT1 expression in undifferentiated human MSCs. Undifferentiated MSCs were seeded (third passage) for 24 h on cover slips, serum starved and treated with 10 ng/mL recombinant IL-10 or remained untreated (control). Subsequently, the cover slips with MSCs were immunolabelled either with STAT3 (**A_1_**–**A_4_**) or STAT1 (**B_1_**–**B_4_**) specific antibodies and Alexa-Fluor^®^488 coupled secondary antibodies (green). As a staining control the primary antibody was omitted (**C_1_**–**C_4_**). Cell nuclei were counterstained using DAPI (blue). The cytoskeleton of the MSCs (also in the staining controls) is depicted using phalloidin-555 staining. A representative experiment of 2 tests with cells of 2 different donors is shown by confocal laser scanning microscopy. Scale bar = 100 µm.

The MSCs showed immunoreactivity of STAT3. In some cells of the population a translocation of STAT3 in response to the short time treatment with IL-10 was detectable (Figure 5). Cells depicted only a faint STAT1 immunoreactivity and very few cells revealed nuclear STAT1 staining in response to IL-10 treatment.

### 2.4. Gene Expression of Chondrogenic Differentiated MSCs under the Influence of IL-10 and TNFα in High-Density (H-D) Culture

**Figure 6:**
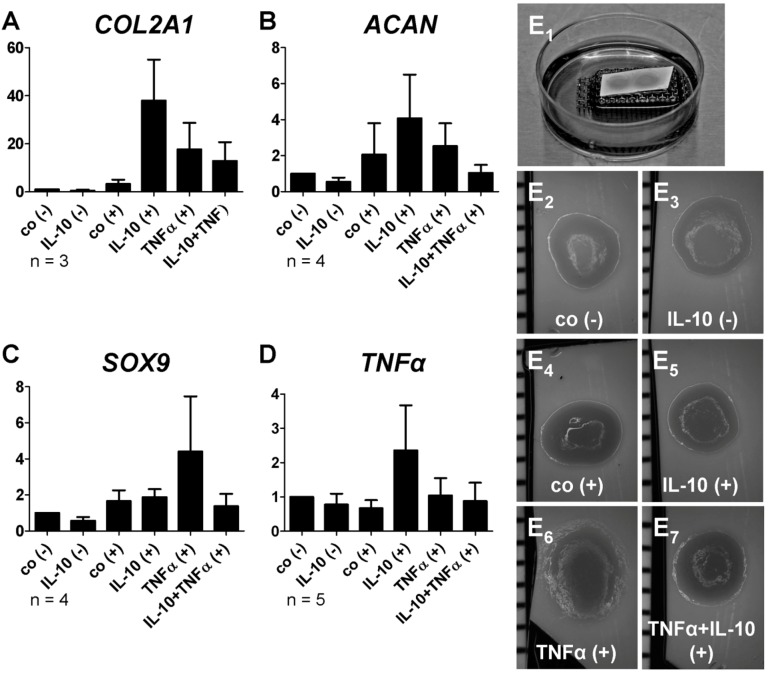
Relative gene expression of cartilage markers in H-D cultures after 14 days of differentiation and 7 days cytokine stimulation. The H-D cultures were differentiated for 14 days and stimulated during the last 7 days with either IL-10 or TNFα alone, or in combination (each 10 ng/mL). *COL2A1* (**A**, *n* = 3) and *ACAN* (**B**, *n* = 4) encodes main structural components of articular cartilage; *SOX9* (**C**, *n* = 4) encodes one of the most important transcription factors during chondrocyte differentiation, whereas *TNFα* (**D**, *n* = 5) encodes a pro-inflammatory cytokine; (**E_1_**–**E_7_**) Representative macroscopic images of the 14 day old H-D cultures after treatment. Data was normalized. (**A**–**C**) The number of experiments did not allow approving a Gaussian distribution of the data. Therefore, a Wilcoxon signed rank test was performed. *p* < 0.05; (**D**) A Gaussian distribution could be determined for the data hence one-sample-*t*-test, one way ANOVA and Bonferoni post test were used. *n* = 3–5 independent experiments with cells of different donors were performed. Co, control; (−) undifferentiated; (+) differentiated.

Neither IL-10 nor TNFα inhibited the chondrogenic gene expression during chondrogenic differentiation of MSC. Moreover, in most of the investigated samples, IL-10 had a slightly stimulatory, but not significant effect on the type II collagen, aggrecan and even TNFα expression when compared with the respective undifferentiated and differentiated controls (Figure 6A–D). Despite not reaching the significance level, TNFα had also inductive effects on *COL2A1*, *SOX9*, *ACAN* and *TNFα* gene expression. The shape and size of the cultures revealed no major differences (Figure 6E_1_–E_7_).

### 2.5. Histological Structure and Type II Collagen Expression of MSCs in H-D Culture

**Figure 7:**
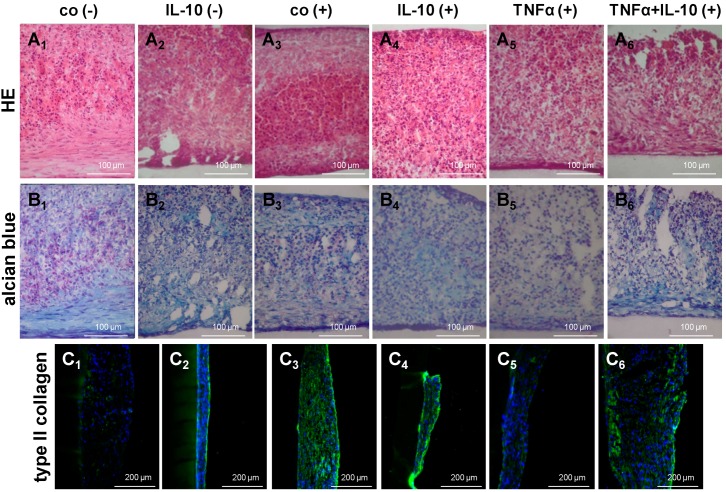
Histology and type II collagen synthesis in chondrogenic differentiated MSCs under the influence of cytokines in H-D cultures. The H-D cultures were differentiated for 14 days and stimulated during the last 7 days with either IL-10 or TNFα alone, or in combination (each 10 ng/mL). HE (**A_1_**–**A_6_**) and alcian blue (**B_1_**–**B_6_**) stainings were performed; Additionally, type II collagen was immunolabelled (green, **C_1_**–**C_6_**). Cell nuclei were counterstained using DAPI (blue). Co, control; (−) undifferentiated; (+) differentiated. Scale bars = 100 µm (**A_1_**–**A_6_** and **B_1_**–**B_6_**), 200 µm (**C_1_**–**C_6_**).

Histological structure of H-D culture under the different treatment conditions was firstly visualized using HE staining (Figure 7A_1_–A_6_). In most cultures the bottom and top cell layers revealed more or less elongated cells whereas the middle of the culture consisted of round cells. Irrespective of cell layer and treatment all cells were embedded into a fibril-rich and alcian blue positive ECM which suggested a substantial content of sulfated glycosaminoglycan (Figure 7B_1_–B_6_). However, cultures treated with TNFα either alone or in combination with IL-10 revealed a looser consistency of the ECM. The ECM of chondrogenically differentiated MSC cultures contained sulfated glycosaminoglycan and type II collagen. There was a slightly higher type II collagen fluorescence intensity in the chondrogenic 3D cultures untreated or treated with IL-10 that correlated with the gene expression results (Figure 7).

### 2.6. Chondrogenic Gene Expression and Histology of Chondrogenic Differentiated MSCs in H-D and Polyglycolic Acid (PGA) Culture

**Figure 8:**
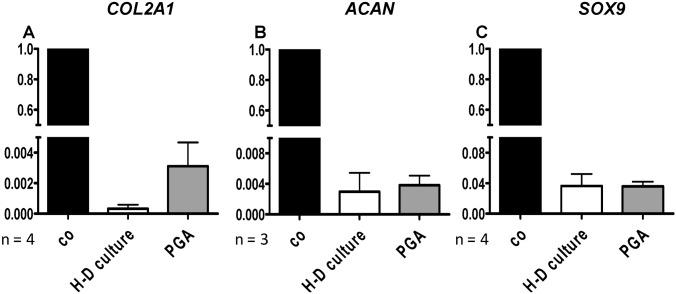
Relative gene expression of chondrogenic differentiated MSCs in H-D culture and on PGA scaffolds compared with freshly isolated, non-cultured articular chondrocytes (co).MSCs (passage 4–6) underwent chondrogenic differentiation for 14 days. (**A**) *COL2A1*; (**B**) *ACAN*; (**C**) *SOX9*. *n* = 3–4 independent experiments with cells of different donors were performed. Data was analyzed using the Wilcoxon signed rank test, Kruskal Wallis and Dunns post test.

**Figure 9:**
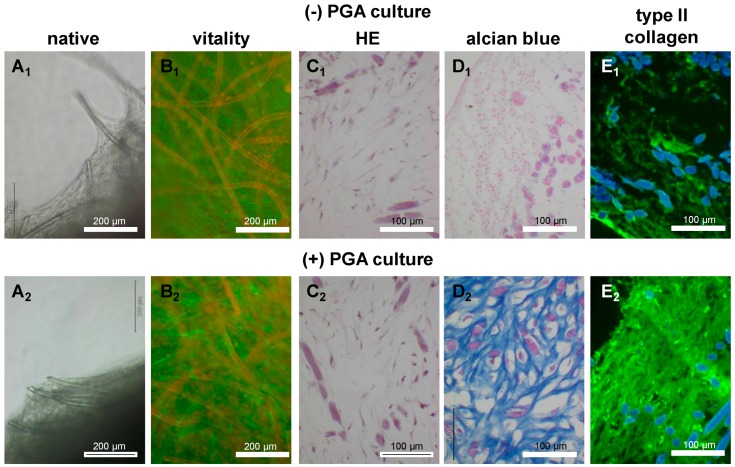
Vitality, histology and type II collagen expression of chondrogenically differentiated and undifferentiated MSCs on PGA scaffolds. Undifferentiated (−) and chondrogenically differentiated (+) MSCs were cultured in PGA. (**A_1_**,**A_2_**) Invert microscopical pictures (“native”) of undifferentiated and chondrogenically differentiated MSCs in PGA, (**B_1_**,**B_2_**) MSCs stained with FDA/PI, dead cells and PGA fibers are red and viable cells are green, (**C_1_**,**C_2_**) HE, (**D_1_**,**D_2_**) alcian blue or immunolabelled for type II collagen (**E_1_**,**E_2_**). In the HE and alcian blue staining the fibers and cell nuclei are stained violet. Cell nuclei were counterstained using DAPI (blue). Scale bars = 200 µm (**A_1_**–**B_2_**), 100 µm (**C_1_**–**E_2_**).

To answer the question whether MSCs could undergo chondrogenic differentiation in scaffold culture in a similar manner to the H-D culture, both 3D culture types were directly compared concerning their gene expression profile. Non cultured human articular chondrocytes served as controls. Chondrogenic differentiated MSCs expressed typical cartilage markers, such as type II collagen, aggrecan and sox9 in H-D culture and PGA scaffold culture (Figure 8A–C). However, gene expression of cartilage markers was inferior in chondrogenic differentiated MSCs cultured in both 3D culture systems compared with freshly isolated human articular chondrocytes. Further, despite the difference was not significant, the expression of type II collagen was higher in PGA compared with H-D culture (Figure 8A). The expression level of aggrecan and sox9 revealed no major differences in both culture systems (Figure 8B,C). The PGA seeded for 14–21 days with chondrogenically induced or non induced MSCs were analyzed for cell vitality, histology and protein expression of type II collagen. MSCs adhered on the PGA and formed cell-matrix sails between the PGA fibers (Figure 9A_1_,A_2_). The majority of MSCs cultured for 14 days on PGA scaffolds survived irrespectively whether chondrogenically induced or not (Figure 9B_1_,B_2_).

Not induced and chondrogenically induced chondrocytes cultured in H-D cultures produced an ECM which contained cartilage-specific sulfated glycosaminoglycans (Figure 9C_1_–D_2_) and type II collagen (Figure 9E_1_,E_2_). However, the synthesis of cartilage-specific glycosaminoglycans and type II collagen was inferior in undifferentiated cells (Figure 9D_1_–E_2_).

## 3. Discussion

Cartilage defects, degenerative or traumatic, remain one of the major problems in orthopedics, orthopedic surgery and regenerative medicine [26]. Chondrogenically differentiated MSCs could represent a therapeutic option as proposed previously [27]. The aim of this study was to determine the effect of particular anti- and pro-inflammatory cytokines (IL-10, TNFα) on the chondrogenesis of pre-differentiated MSCs.

In our study, we were able to demonstrate that bone marrow derived MSCs (BM-MSCs) from all donors investigated expressed typical surface molecules for MSCs. The MSC characterization revealed that the isolated, plastic adherent cell population contained nearly no blood cells such as lymphocyte subpopulations and macrophages discernible by CD3-, CD4- and CD8- as well as CD14-negativity. Most of the MSCs were CD29, CD44 and CD90 positive [28,29]. When characterizing MSCs, BM-MSCs were negative for CD34 [28,30,31]. However, the expression of this marker can be influenced by the origin and passage of stem cells, for instance adipose-derived stem cells at early passages may express CD34 [32,33]. This marker is also typical for the hematopoietic stem cells [28]. Accordingly, in the present study, a high rate of cells negative for the hematopoietic marker CD34 became evident. As far as the CD106 is concerned, the expression of this marker differs among the donors and the origin of the MSCs [28,34,35,36]. The expression of CD106 varied between different donors with the mean of 44.13% ± 28.8% in our study.

Further MSCs expressed IL-10 and the IL-10Rα. IL-10 and/or TNFα did not inhibit the chondrogenic differentiation of MSC. The MSCs expressed STAT3 which is the main downstream transcription factor in IL-10 signaling [37]. Some cells of the population revealed an enhanced STAT3 expression and translocation of STAT3 in response to short time IL-10 exposition suggesting their sensibility for IL-10. Sensibility for IL-10 has already been demonstrated by Jung *et al.* (2013) in MSC micromass cultures [37]. In contrast, STAT1, which can also be induced by IL-10, was only weakly expressed by the MSCs. Furthermore, in most of the investigated samples, IL-10 had a slightly stimulatory effect on the expression of cartilage markers when compared with the respective controls. This observation is in agreement with a study of Jung *et al.* who demonstrated a role for IL-10 in endochondral bone formation stimulating chondrocyte proliferation and hypertrophic differentiation [37]. These cells were able to differentiate into different cell lineages, indicating the multipotent stem cell character of the isolated cells. After chondrogenic differentiation, MSCs cultured in both H-D and PGA culture systems expressed typical cartilage markers. Every biomaterial implanted in the human body may potentially cause an inflammation or infection followed by a rejection. Therefore, as some authors suggest [18], we investigated also the H-D cultures, a culture system which remains mostly unaffected by the influence of a biomaterial.

We found almost no difference between these two culture systems in the gene expression of *ACAN* or *SOX9*. However, the expression of the type II collagen was higher in PGA cultures but the difference did not reach the significance level. It could be assumed that the diffusion in the scaffold culture is easier compared with the H-D culture which is maintained at the medium–air interface and is much denser. The expression profile of cartilage markers was inferior in chondrogenically induced MSCs in both culture systems compared to freshly isolated non cultured chondrocytes underlining the need for further optimization of chondrogenesis. PGA scaffolds have been implicated as a biocompatible scaffold for cartilage repair, yet further clinical studies are required [21,38,39,40,41,42,43]. PGA scaffolds allow chondrogenic differentiation of MSCs not only *in vitro* but also *in vivo*. The cartilage defects covered with PGA scaffolds have shown more hyaline-like repair tissue in comparison to different surgical techniques as for instance microfracturing. However, in the *in vivo* studies so far available only small numbers of animals were investigated and the analyzed cartilage defects were small [21,22,38,44]. Therefore, the role of PGA scaffolds in cartilage repair requires further investigation. Unlike pellet cultures, MSCs were cultivated in micromasses on a porous cellulose membrane where the cell conglomerate can easily be isolated for analysis. The limitation of this method for a potential *in vivo* research is the fact that a great amount of MSCs is required to form a patch big enough to cover cartilage defects. The success of chondrogenic differentiation of MSCs was donor-dependent in this study. Donor and age dependencies of chondrogenic differentiation have already been observed by others [5,45].

In comparison to a healthy tissue, the osteoarthritic joint shows histological features of cartilage degeneration. These features probably support the migration and chondrogenic differentiation of the MSCs [46,47]. Furthermore, traumatic cartilage damage leads to an inflammatory milieu in the joint. High levels of matrix metalloproteinases (MMP) 1 and 3, IL-1β and TNFα have been detected in patients with posttraumatic cartilage defects. These alterations might affect the healing response. TNFα is supposed to play a key role [48,49,50]. A typical anti-inflammatory cytokine in human chondrocytes is IL-10. It has an anti-apoptotic effect and plays an antagonistic role to TNFα [51]. There exist only spare information about the role and the mechanisms of action of IL-10 in cartilage and under OA conditions. It is supposed to have a protective effect on the cartilage tissue [52]. However, the increase of both, TNFα and IL-10 correlates with the OA progression [53].

The expression of IL-10 has been observed in mesodermal cell types not only in chondrocytes [51] and synovial fibroblasts [54], but also in MSCs of different origins, yet the presence of IL-10R and the effect of this cytokine in BM-MSCs requires further investigation [23,55,56].

Moreover, BM-MSCs are able to induce an IL-10 secretion in other immune cells such as T-cells [57,58]. An important issue is the sensitivity of MSCs for IL-10, which has not been sufficiently addressed so far. The study of Jung *et al.* indicates that chondrogenic precursor cells are responsive to IL-10 [37]. An inflammatory micromilieu in the joint triggered by pro-inflammatory cytokines which were released by synovium-derived leukocyte subpopulations is known to affect chondrogenesis [59]. It has been reported that TNFα may enhance the osteogenic differentiation of MSCs *in vitro* [60,61,62,63]. It may also inhibit myogenic differentiation of the myoblasts [64] and the chondrogenic differentiation of chondrocytes [65,66]. In regard to BM-MSCs, the effect of the inflammation, in particular driven by TNFα has not been thoroughly investigated and certainly has not been fully understood. TNFα is able to induce a joint destruction and it is supposed to block the chondrogenic differentiation of the MSCs via NF-κB activation. Through this pathway transcription of sox9, a crucial transcription factor mediating chondrogenesis, can be blocked [8]. Furthermore, the stimulation of TNFα can inhibit the synthesis of typical cartilage markers such as type II collagen or aggrecan and increase the synthesis of degradative enzymes such as MMPs in the MSCs. Some subtypes of MMPs play a key role during joint cartilage degeneration [67]. Furthermore, TNFα amplifies cytokine expression in MSCs and it is an important regulator of MSC migration [68]. The possibility of TNFα inhibition could be an interesting approach in the therapy of cartilage defects. Interestingly, it has been shown recently that intraarticularly administered MSCs are able to impair the systemic TNFα concentrations underlining their immunomodulatory properties [69]. TNFα inhibitors are already common in the therapy of rheumatoid arthritis [65,70,71].

As shown in the Figure 4, freshly isolated MSC expressed both, IL-10 and its receptor. We could hypothesize that there might exist inductive stimuli modulating IL-10 and IL-10Rα expression which were not further investigated by us. IL-10 and/or TNFα did not inhibit the chondrogenic differentiation of MSCs. In contrast, in this study an inductory effect (not significant) of TNFα on the *SOX9* gene expression by the MSCs was detected. It might depend on time point of analysis and culture system. Taking in account the important influence of the microenvironment Chung and Burdick reported that the chondrogenesis by MSCs depended on the particular biomaterial used for 3D culturing [27]. However, protein expression levels and the nuclear translocation indicative for activation of sox9 which is mandatory for mediating chondrogenesis was not tested. In most of the investigated samples, IL-10 had a slightly stimulatory effect on the type II collagen and aggrecan expression when compared with the respective controls. Higher expression of TNFα under the influence of IL-10 could implicate an interaction between these particular cytokines in MSCs. Cytokines were applied in a concentration of 10 ng/mL, which is supposed to correlate with the physiological concentration in peripheral blood [72]. Our purpose was to investigate the effect under standard conditions. However, to estimate potentially remedial effect of IL-10 on chondrogenic differentiation of MSCs higher concentrations of the applied cytokines should be considered.

## 4. Experimental Section

### 4.1. MSC Isolation

MSCs were isolated from human femoral head spongiosa (obtained from 10 patients between the age of 55 and 88 undergoing joint replacement surgeries of the hip joints) using density gradient centrifugation with biocoll separating solution (Biochrom AG, Berlin, Germany).

The spongiosa of a femoral head was minced and pressed through a sieve. Bone spongiosa fragments were removed and the liquid rest was pressed through a 140 μm pore diameter filter membrane (Millipore, Billerica, MA, USA). To remove the remnants of the particles the isolated cell suspension was washed with phosphate buffered saline (PBS) and centrifuged at 200× *g* in 4 °C for 5 min. The purified pellet was mixed with the biocoll solution (Biochrom AG, Berlin, Germany) and centrifuged at 200× *g* in 4 °C. After 20 min the interphase containing MSCs was extracted, washed with PBS and centrifuged at 200× *g* in 4 °C for 5 min. Subsequently, MSCs were resuspended in stem cell growth medium (Table 1) and seeded in culture flasks for cell expansion (Cell plus culture flask, Sarstedt, Nümbrecht, Germany). The cultivation proceeded at 37 °C, 90% air humidity and 5% CO_2_. The growth medium was changed every 2–3 days.

**Table 1:** Chemical composition of used growth media.

Stem Cell Growth Medium	Concentration
Selenium (Sigma–Aldrich, Munich, Germany)	5 ng/mL
Transferrin (Sigma–Aldrich)	5 µg/mL
Linoleic acid (Sigma–Aldrich)	4.7 µg/mL
Insulin (Sigma–Aldrich)	5 µg/mL
Ascorbic acid (Sigma–Aldrich)	1 µg/mL
Dexamethasone (Sigma–Aldrich)	1 µg/mL
MCDB 201 with l-glutamine solution (Sigma–Aldrich)	34 mL/100 mL
Dulbecco’s modified Eagle’s medium (Biochrom AG)	51 mL/100 mL
Fetal calf serum (FCS, Biochrom AG)	15 mL/100 mL
Streptomycin (Biochrom AG)	50 IU/mL
Penicillin (Biochrom AG)	50 IU/mL
**Chondrocytes Growth Medium**	**Concentration**
Ham’s F-12/Dulbecco’s modified Eagle’s medium supplemented with 25 μg/mL ascorbic acid (Sigma–Aldrich)	1000 mL
Streptomycin (Biochrom AG)	50 IU/mL
Penicillin (Biochrom AG)	50 IU/mL
Amphotericin B (Biochrom AG)	2.5 μg/mL
Essential amino acids (Biochrom AG)	1 mL/100 mL
Fetal calf serum (Biochrom AG)	1 mL/100 mL
**Lipogenic Medium**	**Concentration**
Indomethacin (Sigma–Aldrich)	2 µL/mL
Isobutyl-1-methylxanthine (Sigma–Aldrich)	1 µL/mL
Rosiglitazone (Sigma–Aldrich)	1 µL/mL
Insulin (Sigma–Aldrich)	4 µL/mL
Dexamethasone (Sigma–Aldrich)	1 µL/mL
Fetal calf serum (FCS, Biochrom AG)	0.1 mL/mL
Streptomycin (Biochrom AG)	50 IU/mL
Penicillin (Biochrom AG)	50 IU/mL
HEPES (Biochrom AG)	25 µL/mL
Dulbecco’s modified Eagle’s medium with 3.7 g/L NaHCO_3_ and 4.5 g/L glucose (Biochrom AG)	10 mL
**Osteogenic Medium**	**Concentration**
Dexamethasone (Sigma–Aldrich)	1 µL/mL
Glycerol-3-phosphate (Sigma–Aldrich)	10 µL/mL
Ascorbic acid (Sigma–Aldrich)	2 µL/mL
HEPES (Biochrom AG)	25 µL/mL
Streptomycin (Biochrom AG)	50 IU/mL
Penicillin (Biochrom AG)	50 IU/mL
**Chondrogenic Medium**	**Concentration**
l-Glutamine (Biochrom AG)	10 µL/mL
HEPES (Biochrom AG)	25 µL/mL
Sodium pyruvate (Sigma–Aldrich)	10 µL/mL
Dexamethason (Sigma–Aldrich)	0.1 µL/mL
Ascorbic acid (Sigma–Aldrich)	1.7 µL/mL
Prolin (Sigma–Aldrich)	1 µL/mL
ITS+1 (Sigma–Aldrich)	10 µL/mL
Streptomycin (Biochrom AG)	50 IU/mL
Penicillin (Biochrom AG)	50 IU/mL
TGF-β1 (Pepro Tech GmbH, Hamburg, Germany)	10 ng/mL
Dulbecco’s modified Eagle’s medium with 3.7 g/L NaHCO_3_ and 4.5 g/L glucose (Biochrom AG)	10 mL

### 4.2. Chondrocyte Isolation

Freshly isolated non cultured human articular chondrocytes served as a positive control. Primary human articular chondrocytes and MSCs were isolated in accordance with the institutional ethical committee of the Charité-University Medical School Berlin, Campus Benjamin Franklin (Berlin, Germany; Register ID: EA4 024 09; 27 July 2009). Human femoral head articular cartilage chips were obtained from 3 female patients between the age of 83 and 90 undergoing joint replacement surgery for femoral neck fractures. Each surgery was performed within 24 h after the trauma. Chips were cut into small slices followed by digestion with pronase deriving from *Streptomyces griseus* at 20 mg/mL (7 U/mg, Roche Diagnostics, Mannheim, Germany) in Ham’s F-12/Dulbecco’s modified Eagle’s medium (50/50, Biochrom-AG, Berlin, Germany) for 30 min at 37 °C and subsequently with collagenase NB5 deriving from *Clostridium histolyticum* at 1 mg/mL (Serva, Heidelberg, Germany) in chondrocyte growth medium (Table 1). Isolated chondrocytes were resuspended in chondrocyte growth medium containing 10% FCS and seeded in culture flasks (Cell plus culture flask).

### 4.3. MSC Characterisation Using Flow Cytometry

Subsequently to the isolation, approximately 3 × 10^6^ MSCs from each donor were rinsed in PBS followed by a fixation with 4% paraformaldehyde (PFA) solution (Santa Cruz Biotechnology, Inc., Santa Cruz, CA, USA) for 15 min. To characterize the cell type, fixed MSCs were washed with PBS, centrifuged at 400× *g* and labelled with the following mouse anti-human antibodies: CD3, CD4, CD8, CD14, CD29, CD34, CD44, CD90 and CD106. The solution with primary labelled antibodies was diluted 1:200 in PBS and the solution with unlabelled antibodies 1:20 in PBS. Fifty micro liters of each diluted antibody solution was added to approximately 2 × 10^5^ MSCs. The incubation lasted for 30 min at room temperature (RT). Afterwards, the MSCs were washed in PBS and centrifuged at 400× *g.* The supernatants were decanted and the probes were suspended in FACS-buffer consisting of 1× PBS, 1% bovine serum albumin (BSA, Carl Roth GmbH, Karlsruhe, Germany) and 0.01% sodium acid. The unlabeled primary antibodies were detected with appropriate secondary immunophor-labelled secondary antibody solution, diluted 1:200 in PBS, for 30 min at RT. All probes were washed in PBS and centrifuged at 400× *g*. The supernatants were decanted and the probes were suspended in FACS-buffer for analysis. All specifications of used antibodies are listed in (Table 2).

All samples were measured using FACS calibur flow cytometer (BD Bioscience, San Jose, CA, USA). For the analysis of the results FlowJo 7.0 (Tree Star Inc., Ashland, OR, USA) was used.

### 4.4. Chondrogenic, Adipogenic and Osteogenic Differentiation of MSCs

Four H-D cultures from each donor underwent a chondrogenic differentiation that took 14 days. To confirm the multipotency of the MSCs, additionally a chondrogenic, adipogenic and osteogenic differentiation was performed in monolayer culture.

Subsequently to the cultivation process, 20 µM azacytidine (Sigma–Aldrich, Munich, Germany) was added to the growth medium for 24 h. After 24 h approximately 6 × 10^4^ MSCs were cultured as monolayer on cover slips in one well of a 6 well plate. Each well was incubated with 2 mL adipogenic or osteogenic medium (Table 1). The adipogenic medium was changed every two days and after five days the MSC growth medium was added for two days. Two thirds of the osteogenic medium was changed every two or three days. The cover slips with differentiating MSCs were analyzed after 14, 21 and 28 days. To determine the osteogenic differentiation we used von Kossa staining and for the adipogenic differentiation oil red staining (Figure 3).

**Table 2:** Antibodies to surface markers used for flow cytometry and immunofluorescence labeling.

Primary Antibody	Secondary Antibody
CD3, mouse anti-human CD3 (mouse IgG2a), *r*-phycoerythrin conjugate (Caltag, Buckingham, UK)	none
CD4, mouse anti-human CD 4 (mouse IgG2a), fluorescein (FITC) conjugate (Caltag)	none
CD8, mouse anti-human CD 8 (mouse IgG2a), fluorescein (FITC) conjugate (Caltag)	none
CD14, mouse anti-human CD14 antigen (mouse IgG 2a), fluorescein (FITC) conjugate (Invitrogen, Carlsbad, CA, USA)	none
CD29, mouse anti-human Integrin β1 monoclonal antibody (mouse IgG1) (Millipore, Billerica, MA, USA)	Donkey F(ab)2 Fragment-anti-mouse-APC (Dianova, Hamburg, Germany)
CD34, mouse anti-human CD34 (mouse IgG1, k), allophycocyanin (APC) conjugate (BD Pharmingen, Franklin Lakes, NJ, USA)	none
CD44, mouse anti-human CD 44 antibody (mouse IgG 2a) (Cell signaling, Cambridge, UK)	Donkey F(ab)2 Fragment-anti-mouse-APC (Dianova, Hamburg, Germany)
CD90, mouse anti-human CD90 (mouse IgG1, k), fluorescein (FITC) conjugate (BD Pharmingen)	none
CD106, mouse anti-human VCAM-1 monoclonal antibody (mouse IgG1) (Chemicon, Billerica, MA, USA)	Donkey F(ab)2 Fragment-anti-mouse-APC (Dianova)
Type II collagen, rabbit anti-human polyclonal antibody (Acris Antibodies, Herford, Germany)	Alexa-Fluor^®^488, donkey-anti-rabbit (Invitrogen)
IL-10, rabbit anti-human polyclonal antibody (tebu bio GmbH, Le-Perray-en-Yvelines, France)	Alexa-Fluor^®^488, donkey-anti-rabbit (Invitrogen)
IL-10-Receptor-α, mouse anti-human monoclonal antibody (Sigma–Aldrich)	Alexa-Fluor^®^488, donkey-anti-mouse (Invitrogen)
STAT1, rabbit anti-human monoclonal antibody (Cell Signaling)	Alexa-Fluor^®^488, donkey-anti-mouse (Invitrogen)
STAT3, rabbit anti-human monoclonal antibody (Cell Signaling)	Alexa-Fluor^®^488, donkey-anti-mouse (Invitrogen)

### 4.5. MSCs in H-D Culture and Cultured in Non-Woven PGA Scaffolds

One million eight hundred thousand MSCs, expanded at least until passage 4–6 to achieve sufficient cell numbers, were introduced in scaffold-free H-D culture.

For each donor, one 6-well-plate was prepared for six scaffold-free H-D cultures. In each well one metal grid was placed and covered with a cellulose acetate filter (pore size 0.2 µm, Sartorius AG, Göttingen, Germany) on the top of it. MSCs were detached from culture flasks using 0.05% trypsin/0.02% EDTA (Biochrom AG), washed with PBS and centrifuged in falcon tubes twice. Eight to ten micro liters of the pure MSC cell pellet was transferred on each of the filter membrane. To avoid direct contact between growth medium and the 3D culture, only 1.8 mL of medium per well was added. The cultivation proceeded at 37 °C, 90% air humidity and 5% CO_2_. Additionally, about 4 × 10^6^ MSCs per donor were transferred to non-woven PGA scaffolds (1 × 1 × 0.11 cm) which were cultured in the insert of a two chamber system (pore size 0.4 µm, Beckton–Dickinson, Franklin Lakes, NJ, USA). The scaffolds remained in alginate coated 6-well plates with 3 mL of chondrogenic induction or stem cell growth medium (control) per well. The cultivation under chondrogenic or non-chondrogenic conditions proceeded at 37 °C, 90% air humidity and 5% CO_2_. The 14-day-old scaffolds were rinsed in PBS and then incubated in fluorescein diacetate (FDA) (3 µg/mL dissolved in acetone (stock solution), Sigma–Aldrich and further diluted 1:1000 in PBS (working solution)) for 15 min at 37 °C, rinsed three times with PBS before being counterstained with propidium iodide (PI, Sigma–Aldrich) solution (1 mg/mL dissolved in PBS (stock solution), Sigma–Aldrich, further diluted 1:100 in PBS (working solution)) for 1 min in the dark at RT. The green or red fluorescence was visualized using fluorescence microscopy.

### 4.6. Differentiation of MSCs and Stimulation with Cytokines

Four H-D cultures were chondrogenic differentiated for seven days in chondroinductive medium (Table 1). Two additional H-D cultures remained undifferentiated and were treated only with MSC growth medium.

For the next 7 days the chondrogenic differentiated H-D cultures were stimulated with 10 ng/mL recombinant IL-10, TNFα (both: Peprotech GmbH, Hamburg, Germany), TNFα with IL-10, or remained untreated. One undifferentiated H-D culture was stimulated with 10 ng/mL recombinant IL-10 and one culture remained untreated.

### 4.7. Histological Staining Procedures

For histological staining procedures cryo-sections (thickness: 7 µm), or cover slips were used. For Haematoxylin & Eosin (HE) staining sections were incubated for 4 min in Harris haematoxylin solution (Sigma–Aldrich) rinsed in water and counterstained for 4 min in eosin (Carl Roth GmbH, Karlsruhe, Germany).

For alcian blue (AB) staining, the sections or cover slips were incubated for 3 min in 1% acetic acid and then stained 30 min in 1% AB (Carl Roth GmbH). Subsequently, they were rinsed in 3% acetic acid. Counterstaining of cell nuclei was performed using nuclear fast red aluminum sulfate solution (Carl Roth GmbH) for 5 min.

For von Kossa staining, the sections, or the cover slips were incubated for 5 min in methanol, rinsed in water and then stained 30 min in 1% silver nitrate. After rinsing in water, a reduction with 5% sodium bicarbonate was performed for 7 min. Counterstaining of cell nuclei was performed using nuclear fast red aluminum sulfate solution (Carl Roth GmbH) for 5 min.

For oil red staining, the cover slips were incubated for 20 min in 4% PFA, rinsed in PBS and then stained 30 min in 60% oil red solution (Sigma–Aldrich).

Finally, all sections were rinsed with aqua dest, and subsequently dehydrated in an ascending alcohol series. Then, the sections were embedded with Entellan (Merck, Darmstadt, Germany). All slices were analyzed by light microscopy (Axioskop 40 microscope: Zeiss Jena, Jena, Germany). Imaging of the sections were achieved using an Olympus digital camera XC30 (Olympus Soft Imaging Solutions GmbH, Muenster, Germany).

### 4.8. Gene Expression Analysis Using RTD-PCR

Gene expression was determined using RTD-PCR. MSCs were cultured for at least 14 days in 3D culture. MSC total RNA was isolated using MasterPure™ Plant RNA Purification Kit (MasterPure Plant, RNA Purification-Kit, Epicentre, Biotechnologies, Madison, WI, USA). RNA quantity and quality was evaluated with the RNA 6000 Nano assay (Agilent Technologies, Santa Clara, CA, USA). Equal amounts of total RNA (500 ng in a volume of 20 µL) were reverse transcribed using the Qiagen QuantiTect reverse transcription Kit (Qiagen, Hilden, Germany) according to the manufacturer’s instructions. One micro liter aliquots containing 16.7 ng of cDNA were amplified by RTD-PCR in a 20-µL reaction mixture using specific primer pairs for *COL2A1*, *ACAN*, *SOX9*, *TNFα* and the house-keeping gene *ACTB* (all: Applied Biosystems, Foster City, CA, USA). Assays were performed using the TaqMan Gene Expression Assay (Applied Biosystems) or the Quantitec Gene Expression Assay (Qiagen) in an Opticon 1 Real-Time-Cycler (Opticon™ RTD-PCR, Bio-Rad, Hercules, CA, USA) according to the manufacturer’s protocols. We performed for each primer (Table 3) used in this study an efficiency testing using a linear regression analysis using MSC cDNA. Relative amounts of mRNA expression for the gene of interest, and the *ACTB* were calculated using the ΔΔ*C*_t_ method [73].

**Table 3:** Sequences of the primers used in the present study.

Gene (Symbol)	NCBI Gene Reference	Length	Manufacturer
*β-actin* (*ACTB*)	NM_001101.2	171	ABI^®^ *
*aggrecan* (*ACAN*)	NM_013227.2	93	ABI^®^ *
*sox9* (*SOX9*)	NM_000346.2	102	ABI^®^ *
*TNFα* (*TNFα*)	NM_000594.2	80	ABI^®^ *
*β-actin* (*ACTB*) (5'–3')	TGGGACGACATGGAGAA/GAAGGTCTCAAACATGATCTGG	146	Qiagen^®^
*type II collagen* (*COL2A1*)	NM_001844, NM_033150	142	Qiagen^®^

* ABI, Applied Biosystems^®^ (Life Technologies™, Carlsbad, CA, USA).

### 4.9. Immunolabelling

Undifferentiated MSCs isolated from three different donors were cultured for 48 h on cover slides. Cryo-sections of 14-day-old 3D cultures and cover slides with cells were fixed with 4% PFA solution for 15 min before rinsed in Tris buffered saline (TBS: 0.05 M Tris, 0.015 M NaCl, pH 7.6). Sections were subsequently blocked with protease-free donkey serum ((Merck Millipore), 5% diluted in TBS) for 30 min at RT, rinsed and incubated with the anti-type II collagen antibody in a humidifier chamber overnight at 4 °C. Cover slides were immunolabelled with anti-IL-10, IL-10Rα, STAT1, STAT3, CD3, CD4, CD8, CD14, CD29, CD34, CD44, CD90, CD106 antibodies. Sections and cover slides were subsequently washed with TBS before incubation with Alexa-Fluor^®^488 secondary antibodies for 30 min at RT. STAT3 and STAT1 immunolabelling was combined with phalloidin-CruzFluor555-staining (diluted 1:200 in blocking buffer, Santa Cruz Biotechnology, Biotechnology, Santa Cruz, CA, USA) to depict the cytoskeleton. Negative controls included omitting the primary antibody or using human IgG as primary antibody during the staining procedure. Cell nuclei were counterstained using 4',6-diamidino-2-phenylindole (DAPI) (0.1 µg/mL, Roche Diagnostics, Mannheim, Germany). Labelled sections were rinsed several times with TBS, embedded with Fluoromount G (Southern Biotech, Biozol Diagnostica, Birmingham, AL, USA) and examined using fluorescence microscopy (Axioskop 40) or confocal laser scanning microscopy (SPE-II, Leica, Wetzlar, Germany). Images were taken using the XC30 camera. All specifications of used antibodies are listed in (Table 2).

### 4.10. Statistical Analysis

All values were expressed as mean with standard deviation. Kolmogorov-Smirnov test was used to detect the presence of Gaussian distribution. Data of experiments where the Gaussian distribution could not be determined due to only *n* = 3–4 was analyzed using the Wilcoxon signed rank test, Kruskal Wallis and Dunns post test (GraphPad Prism 5, GraphPad Software Inc., San Diego, CA, USA). Data for which a Gaussian distribution could be proven were analyzed using one-sample-*t*-test, one way ANOVA and Bonferoni post test. Statistical significance was set at a *p* value of ≤0.05.

## 5. Conclusions

In summary, independent of the 3D culture system used for differentiation, the expression of chondrogenic markers was lower in differentiated MSCs compared with freshly isolated chondrocytes. Chondrogenic differentiated MSCs expressed *COL2A1*, *ACAN* and *SOX9*. An improved understanding of IL-10 and TNFα involvement in chondrogenesis could be a key issue to optimize cell-based joint repair strategies, so the influence of IL-10 and TNFα on MSC differentiation requires further investigation.
